# Whip It Good: A Case of Vitamin B12 Deficiency and Subacute Combined Degeneration of the Spinal Cord

**DOI:** 10.7759/cureus.74620

**Published:** 2024-11-27

**Authors:** Idean A Pourshams, Manik Arora, Santosh Nimkar, Pradeep Kumbham

**Affiliations:** 1 Internal Medicine, University of Arkansas for Medical Sciences, Fayetteville, USA; 2 Neurology, Osteopathic Medical School, Kansas City University, Kansas City, USA; 3 Neurology, Mercy Hospital Northwest Arkansas, Arkansas, USA

**Keywords:** causes of vitamin b12 deficiency, gait ataxia, methylcobalamin, nitrous oxide abuse, subacute combined degeneration

## Abstract

A 40-year-old male patient with no significant past history presented to the emergency room with bilateral upper and lower extremity numbness and difficulty walking for three weeks. MRI of the thoracic spine revealed cord signal abnormalities in the dorsal columns consistent with selective degeneration. This was congruent with the patient's presentation and symptoms of myelopathy with dorsal column involvement along with peripheral polyneuropathy. Nitrous oxide inhalation was the culprit, causing acute vitamin B12 deficiency and subsequent myeloneuropathies.

## Introduction

Subacute combined degeneration (SCD) of the spinal cord is a neurological disorder characterized by progressive demyelination and loss of neurons within the spinal cord, particularly affecting the posterior and lateral columns. This demyelination process is closely associated with impaired synthesis of myelin, the protective sheath around nerve fibers. Myelin is crucial for rapid and efficient nerve impulse conduction, and its integrity is maintained largely by vitamin B12, an essential nutrient [1]. Without sufficient B12, myelin cannot be synthesized properly, leading to degenerative changes in the nervous system, including SCD. Symptoms often progress gradually, causing significant functional impairment if untreated. 

One lesser known factor leading to B12 inactivation and subsequent demyelination in SCD is the inhalation of nitrous oxide, commonly known as "laughing gas." When nitrous oxide is inhaled, it initiates a chain of biochemical reactions that inactivate B12, disrupting the metabolic pathways that support myelin integrity. Chronic exposure to nitrous oxide or high doses can lead to profound B12 deficiency, even in individuals who otherwise maintain a balanced diet. This gas is sometimes used recreationally in the form of “whippets” or “galaxy gas,” where small canisters containing nitrous oxide are inhaled for a brief, euphoric high [2]. However, repeated recreational use can have serious neurological consequences, including SCD [3-6]. 

Clinicians should consider nitrous oxide exposure, particularly through recreational use, when evaluating patients who present with neurological symptoms like sensory deficits, weakness, ataxia, or paresthesia. These symptoms are typical of SCD but may be accompanied by changes in mental status or visual disturbances as the disease progresses. Differential diagnoses should include SCD, especially in younger individuals without traditional risk factors for B12 deficiency. Prompt recognition and diagnosis of nitrous oxide-related SCD are essential, as early intervention with B12 supplementation can halt disease progression and potentially reverse some symptoms.

## Case presentation

A 40-year-old male patient with past medical history of asthma presented to the emergency department with bilateral upper and lower extremity numbness, difficulty walking for three weeks as well as pain and pressure in the abdomen wrapping around to the back. The numbness was progressively worsening, with developing paresthesia, described as electric shock-like sensations when his feet touched any surface with pressure. The abdominal pain had persisted for two days with radiation to the groin. He endorsed constipation for three days, noted to be unusual for the patient. There was no facial weakness, difficulty swallowing, urinary incontinence, shortness of breath, periods of apnea, visual disturbances or speech difficulty. Two months prior to presentation, the patient recovered from COVID after mild flu-like symptoms. He had previously received two doses of the COVID vaccine. He denied any history of tick bites, and he was not taking any prescription or over-the-counter medications. 

Upon presentation to the emergency department, our patient was alert and oriented to time, location, name and situation, speaking clearly without dysarthria. Pupils were equal and reactive to light, extraocular muscles were intact and there was no evidence of nystagmus. There were no facial sensory deficits, or asymmetry of the face with eye closure, smile, shoulder shrug or tongue protrusion. There was no evidence of visuospatial neglect upon confrontation. The patient did not have any bulbar or respiratory muscle weakness. Motor strength was 4 out of 5 on the left lower extremity, 5 out of 5 on the right lower extremity, 5 out of 5 at the shoulders and 3 out of 5 in the hands. The patient endorsed some numbness or diminished sensation on the abdomen in the T8-T10 dermatomal distribution. Babinski testing was down-going bilaterally; however, pressing the reflex hammer upon the plantar surface of the foot elicited severe electrical pain. The patient also complained of numbness above the knee. The patient's hands looked contracted at the fourth and fifth digits. Neck flexion elicited a positive Lhermitte sign with shooting paresthesias down to his feet. The patient did not display any dysmetria with finger-to-nose or heel-to-shin testing. There was no evidence of dysdiadochokinesia. The patient was unsteady when trying to stand, and Romberg sign was positive. 

**Figure 1:**
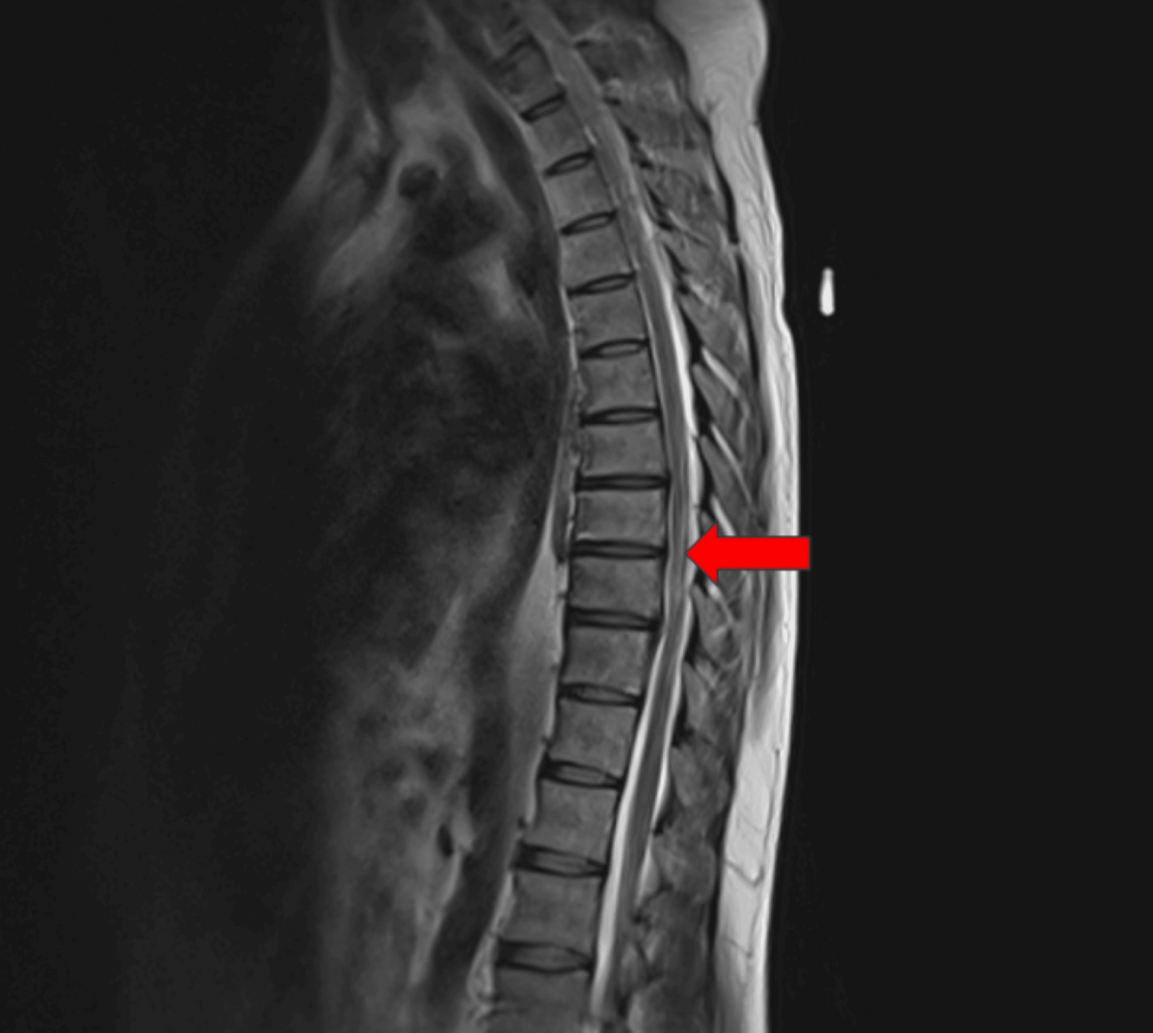
Sagittal view of cord signal abnormalities confined to the dorsal columns is most consistent with selective degeneration secondary to vitamin B12 deficiency. The red arrow indicates the areas of subacute combined degeneration on MRI.

**Figure 2:**
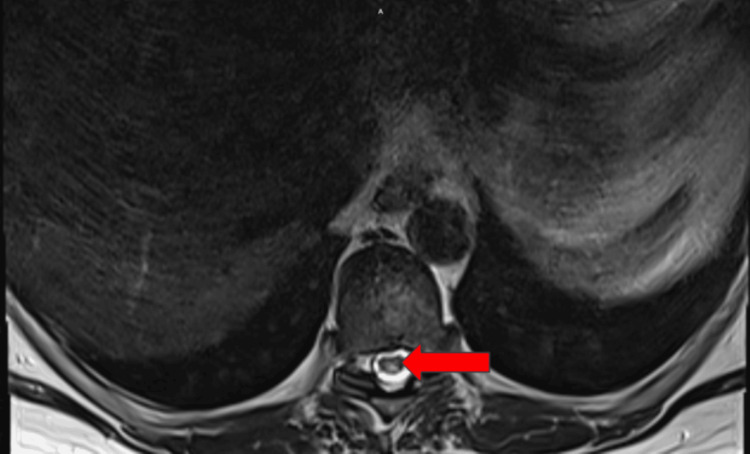
Axial view of cord signal abnormalities confined to the dorsal columns. The red arrow indicates the areas of subacute combined degeneration on MRI.

**Figure 3:**
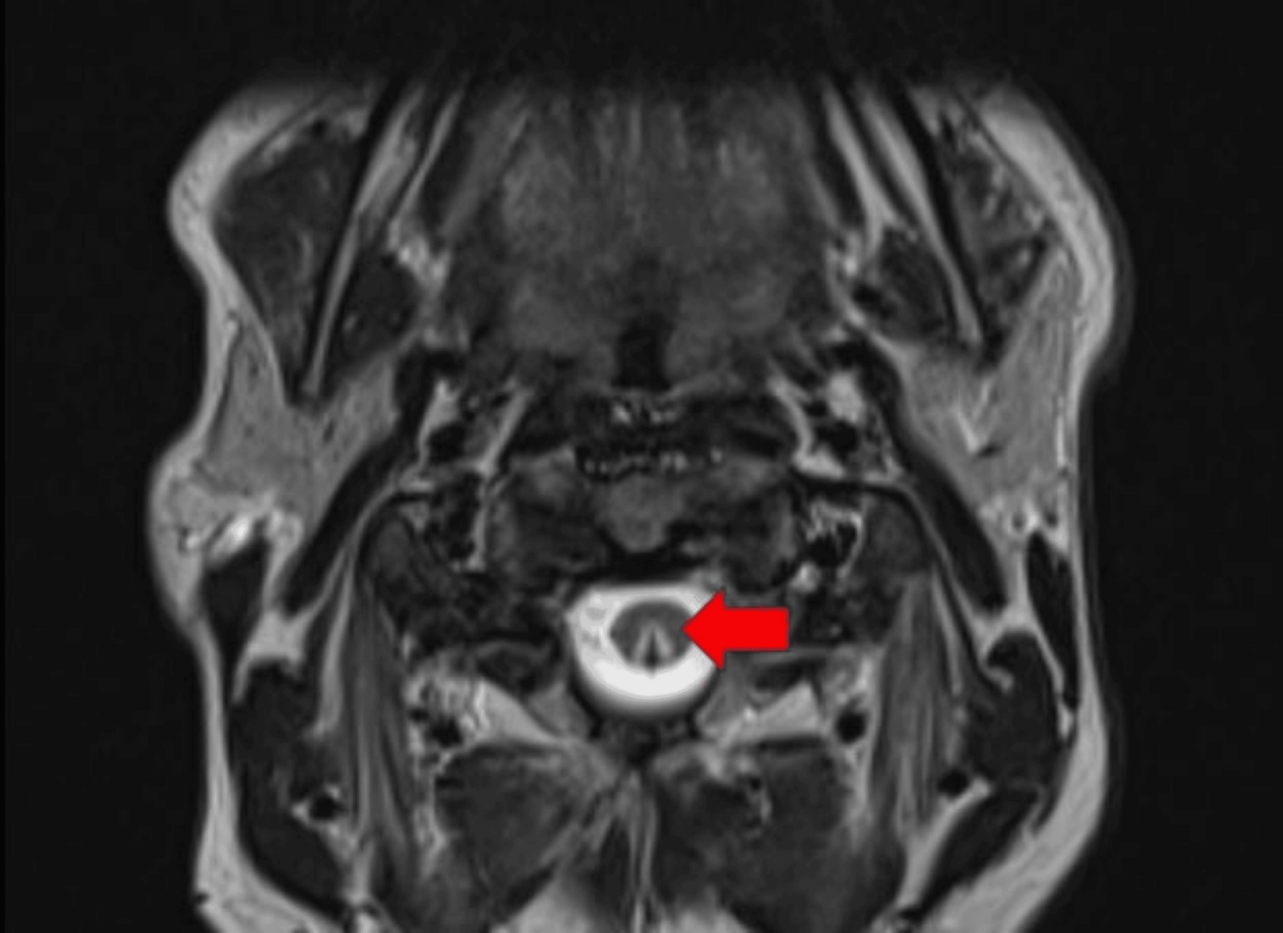
Closer axial view of signal abnormalities confined to the dorsal columns. The red arrow indicates the areas of subacute combined degeneration on MRI.

MRI of the thoracic spine revealed T2 cord signal abnormalities in the dorsal columns consistent with selective degeneration. This was expected and consistent with vitamin B12 deficiency. Other possible differential diagnoses may have included infectious/inflammatory myelitis or acute transverse myelitis, which were ruled out by the distinct findings on imaging. This particular patient also had degenerative disc disease with mild spinal stenosis from T9-T10 and C2-C6. The cervical MRI revealed the quintessential dorsal column finding with T2 hyperintensity in an inverted V pattern (Figures 1-3).

Vitamin B12 deficiency was suspected and its levels were severely low. This was congruent with the patient's presentation and symptoms of myelopathy with dorsal column involvement along with peripheral polyneuropathy. Subacute combined degeneration of the spinal cord was confirmed with cervical and thoracic MRI as seen above. The patient was then treated with vitamin B12, 1 mg IM (intramuscular) daily along with sublingual 2500 mcg daily. Initial lab values are listed below in Table 1.

**Table 1:** Initial Lab Results RBC: Red blood cells; MCH: mean corpuscular hemoglobin; MCHC: mean corpuscular hemoglobin concentration

Parameters	Patient values	Reference range and units
RBC	3.78	4.20-5.80 million/uL
Hemoglobin	13	13.2-17.1 g/dL
MCH	34.4	27.0-30.0 pg
MCHC	34.9	32.0-36.0 g/dL
Vitamin B12	151	200-1100 pg/mL

As we attempted to understand the etiology of the B12 deficiency, the patient denied alcohol abuse and indicated that he followed a diet including meat and dairy products. After one day in the hospital, the patient reported slight improvement subjectively but continued to have tremendous gait difficulty including a positive Romberg sign, ruling out the possibility of the patient going home without rehabilitation. His abdominal pain had slightly improved after a small bowel movement.

We discussed the patient's prognosis. Trying to determine the cause of the vitamin B12 deficiency, we explained that we were going to test the patient for pernicious anemia with intrinsic factor antibodies. We further delved into the patient's use of alcohol and long-term diet looking for any explanation. We inquired of the patient's dental history for the possibility of frequent exposure to nitrous oxide. At this point, the patient reported frequent use of nitrous oxide inhalants for recreational purposes during the last year, with most recent use within one week of presentation. This became the primary suspicion for the cause of the acute vitamin B12 deficiency along with the presenting myeloneuropathies.

With physical therapy, the patient was able to ambulate 80 feet with a rolling walker requiring moderate assistance for balance. He continued to be very ataxic with hyperextension of both knees when loadbearing, slapping his feet onto the floor as expected with loss of proprioception. Despite the patient's persistent request to return home, he understood the need for rehabilitation. The patient's abdominal pain in a bandlike pattern wrapping to his back was likely a further manifestation of subacute combined degeneration of the spinal cord. He continued to improve on hospital day three, regaining strength in all extremities along with improvement of sensation bilaterally in the upper and lower extremities.

## Discussion

Vitamin B12 is an integral cofactor for two essential enzymes related to myelin, including homocysteine methyltransferase and methionine synthase. These enzymes maintain the neuronal sheath. Without vitamin B12, the synthesis of myelin is impaired, resulting in the demyelinating condition of subacute combined degeneration of the spinal cord [1].

Nitrous oxide use as an inhaled anesthetic during surgery can cause activation of methylcobalamin and subsequent oxidation, thereby inactivating vitamin B12. Supplementation is recommended after gastric or bariatric surgery due to intrinsic factor deficiency caused by loss of gastric parietal cells. Resection of the ileum and other malabsorption conditions may also affect vitamin B12 levels. Diet and alcohol use are important to determine the etiology of vitamin B12 deficiency, but it's important to remember that vegan diets may take two to three years before developing B12 deficiency.

N_2_O inactivates methylcobalamin by oxidizing the cobalt ion in Vitamin B12 from a 1+ to a 3+ valence state that can drastically change the effect of B12 on various biochemical pathways [1]. B12 is used as a cofactor for methionine synthase, which converts homocysteine to methionine. This is done by transferring a methyl group to form methionine from methyl-tetrahydrofolate, which becomes tetrahydrofolate to be used for DNA/RNA/protein synthesis. This decrease in methionine production leads to disruption in the production of phospholipids of the myelin sheath [1]. Cobalamin inactivation is also responsible for the increased accumulation of methylmalonic acid by decreasing the function of methylmalonyl-CoA mutase. Both substrates then accumulate within the myelin sheath of the spinal column, leading to degenerative demyelination. It presents as paresthesia and weakness. Homocysteine also accumulates within the myelin sheath and acts upon N-methyl-D-aspartate (NMDA) receptors, leading to overstimulation of the receptor, which causes increased calcium ions in the cytoplasm and increased amounts of reactive oxygen species (ROS) leading to apoptosis [3].

“Whippets,” "galaxy gas" or “laughing gas” are terms used to colloquially refer to nitrous oxide. Originally discovered by Joseph Priestly in 1772, nitrous oxide quickly gained favor as it was found to have anesthetic capabilities in 1844 by a dentist named Horace Wells [4,5]. Since then, nitrous oxide has become a legitimate and widely used sedative and anesthetic in medicine and dentistry.

However, the “euphoric” and “hallucinogenic” effects of nitrous oxide have led to widespread abuse. According to a 2022 survey by the U.S. Substance Abuse and Mental Health Services Administration (SAMHSA), 12,387,000 American adolescents above the age of 12 report illicit use of nitrous oxide (2022 National Survey on Drug Use and Health). Additionally, nitrous oxide is also readily accessible in the form of “whipped cream chargers”, which are packaged and sold worldwide for use as food products. The illicit use involves the transfer of this nitrous oxide from the charger to some air receptacle like a balloon, which is then inhaled by the user [4,5]. Unfortunately, the risk of nitrous oxide abuse is largely unknown to the public. As seen in this case report, the abuse of nitrous oxide can result in B12 deficiency and subacute combined degeneration due to nitrous oxide’s ability to alter the body’s metabolism of B12 [5-11].

Our patient in this case was unaware of the harmful effects of nitrous oxide inhalation. When initially asked about substance use in the emergency department, he denied its use because he did not associate breathing "whipped cream gas" with substance abuse. He did not correlate the severe symptoms of ataxia to nitrous oxide until we were able to fully explain the side effects.

## Conclusions

This case presentation is important to remind ourselves of the possibility of nitrous oxide being used as a recreational drug and its impact on neurological health, specifically its effect on vitamin B_12_. Our patient presented severely ataxic, with foot slapping upon attempting to ambulate. Vitamin B_12_ deficient patients may present with confusion, ataxia or visual deficits. Nitrous oxide canisters are available without sales guidelines for purchase related to food and drink preparation but are termed "whippets" or "galaxy gas" for recreational use. This example also serves to remind health practitioners to remember advocating against the use of harmful substances that our patients may use for euphoria without realizing the consequences. It was only upon further inquiry that we were able to ascertain the true etiology of our patient's presenting symptoms, and we encourage other providers to also diligently gather patient history in a nonjudgmental fashion while also educating patients of the potential neurological damage related to substance abuse. With deeper understanding of the risks, patients may be deterred from such harmful activities.
